# Development of monoclonal anti-PDGF-CC antibodies as tools for investigating human tissue expression and for blocking PDGF-CC induced PDGFRα signalling *in vivo*

**DOI:** 10.1371/journal.pone.0201089

**Published:** 2018-07-27

**Authors:** Hong Li, Manuel Zeitelhofer, Ingrid Nilsson, Xicong Liu, Laura Allan, Benjamin Gloria, Angelo Perani, Carmel Murone, Bruno Catimel, A. Munro Neville, Fiona E. Scott, Andrew M. Scott, Ulf Eriksson

**Affiliations:** 1 Department of Medical *Biochemistry* and Biophysics, Karolinska Institutet, Stockholm, Sweden; 2 Ludwig institute for Cancer Research, Melbourne Austin Branch, Melbourne, Australia; 3 Olivia Newton-John Cancer Research Institute, and School of Cancer Medicine, La Trobe University, Melbourne, Australia; 4 Ludwig Institute for Cancer Research, New York, New York, United States of America; Duke University School of Medicine, UNITED STATES

## Abstract

PDGF-CC is a member of the platelet-derived growth factor (PDGF) family that stimulates PDGFRα phosphorylation and thereby activates intracellular signalling events essential for development but also in cancer, fibrosis and neuropathologies involving blood-brain barrier (BBB) disruption. In order to elucidate the biological and pathological role(s) of PDGF-CC signalling, we have generated high affinity neutralizing monoclonal antibodies (mAbs) recognizing human PDGF-CC. We determined the complementarity determining regions (CDRs) of the selected clones, and mapped the binding epitope for clone 6B3. Using the monoclonal 6B3, we determined the expression pattern for PDGF-CC in different human primary tumours and control tissues, and explored its ability to neutralize PDGF-CC-induced phosphorylation of PDGFRα. In addition, we showed that PDGF-CC induced disruption of the blood-retinal barrier (BRB) was significantly reduced upon intraperitoneal administration of a chimeric anti-PDGF-CC antibody. In summary, we report on high affinity monoclonal antibodies against PDGF-CC that have therapeutic efficacy *in vivo*.

## Introduction

Platelet-derived growth factors (PDGFs) have important functions during development but also in diverse pathological conditions [1]. PDGF-CC is a member of the PDGF family that has been extensively characterized during the past decade [2–6]. Upon PDGF-CC binding, PDGF receptor alpha (PDGFRα) subunits become dimerized, leading to receptor autophosphorylation. This initiates intracellular signalling events triggering responses such as cell proliferation, migration, contraction and survival [7]. In contrast to other PDGF family members, activation of full-length PDGF-CC requires proteolytic cleavage whereby the dimeric conserved growth factor domain (GFD) is released from the N-terminal CUB domain. Activation of latent PDGF-CC by tissue plasminogen activator (tPA) has been characterized in detail and seems largely confined to the CNS [8, 9], while in other tissues, other proteases can activate PDGF-CC [2, 10].

Autocrine and paracrine PDGF signalling is involved in pathogenesis of gliomas, sarcomas, leukemias and epithelial cancers [11, 12]. In addition, PDGFs drive pathological mesenchymal responses in vascular disorders and fibrotic diseases [1]. Further, dysregulation in the PDGF/PDGFR system such as constitutive activation of PDGFRs, or mutations which up/downregulate both ligand and receptor activities, contribute to osteosarcomas, lung carcinomas, gliomas, astrocytomas and medulloblastomas [12, 13]. Apart from PDGF-CC being a potent proto-oncogene that induces malignant transformation of fibroblasts, it also serves as a mitogen for many cell types of mesenchymal origin, including certain stem cells, smooth muscle cells and endothelial cells [7, 9, 14, 15].

The importance of PDGF-CC in tumour biology has recently been emphasized by demonstration of the capacity of PDGF-CC to facilitate tumour growth via recruitment of cancer-associated fibroblasts (CAFs) into the tumour mass, and also by increasing tumour angiogenesis through induction of the angiogenic factor osteopontin [16]. It has also been reported that overexpression of PDGF-CC resulted in liver steatosis, fibrosis, and hepatocellular carcinoma in a *Pdgfc* transgenic mouse model [17] and that PDGF-CC mediates renal interstitial fibrosis [18]. Increased expression of PDGF-CC has additionally been observed in experimental models of heart and lung fibrosis [19, 20]. PDGF-CC has been shown to mediate angiogenic and tumorigenic properties of fibroblasts associated with tumours refractory to anti-VEGF treatment [21]. Notably, in preclinical models of head and neck squamous cell carcinoma, siRNA-mediated reduction of PDGF-CC expression levels reversed resistance to cisplatin and thus improved the therapeutic effect [22].

PDGF-CC has been recognized as a regulator of the blood-brain barrier (BBB) integrity. It has been shown that intraventricular injection of active PDGF-CC protein is sufficient for disrupting the BBB [23]. Moreover, inhibition of the PDGF-CC/PDGFRα axis reduced BBB dysfunction and has beneficial therapeutic effects in animal models of stroke [23–25], spinal cord injury [26], multiple sclerosis [27], traumatic brain injury [28], seizures [29] and amyotrophic lateral sclerosis [30]. Recently, a phase 2 clinical trial in human ischemic stroke subjects treated with thrombolysis and the PDGFRα inhibitor imatinib, showed significant improvements in clinical outcome compared to thrombolysis alone [31].

Molecular tools specific for targeting PDGF-CC signalling are still missing, and we aimed to generate high affinity monoclonal antibodies (mAbs) suitable for functional assessment of PDGF-CC signalling with a potential for therapeutic use [32]. For that purpose we have generated and characterized mAbs against human PDGF-CC and assessed their binding specificities, affinities and complementarity-determining regions (CDR). We demonstrated their ability to neutralize PDGF-CC mediated activation of PDGFRα. We have also investigated the expression pattern of PDGF-CC in human primary tumours and control tissues. Taken together, the properties of our high affinity mAbs indicate that they are potentially useful for the treatment of various PDGF-CC related pathologies in cancer, fibrosis, and CNS diseases.

## Materials and methods

All laboratory health and safety procedures have been complied with in the course of conducting the experimental work reported in this paper.

### Generation of monoclonal antibodies

To produce mAbs against PDGF-CC, we used recombinant human PDGF-CC core domain protein [2]. Immunizations and generation of hybridomas were carried out by the EMBL monoclonal antibody facility, Rome, Italy (http://www.macf.embl.de). 16 hybridomas reacting strongly with PDGF-CC in both ELISA and immuno blots (WBs) were selected. The hybridomas were grown in HM20 medium containing DMEM (Gibco, 41965–039) and supplemented with 20% FBS (Gibco, 10437–036), 50 μg/ml gentamicin (Gibco, 15757–037), 200 mM L-glutamate (Gibco, 25030–024) and 10% HCF (hybridoma cloning factor, BioVeris Corporation, Ca: 210001) at 37°C, with 5% CO_2_. The hybridomas were subcloned by serial dilution and expanded. To purify the mAb IgGs, the expanded cultures were grown in reduced serum media. To avoid unspecific IgG contamination from FCS, we used Ultra-Low IgG FBS (Gibco, 16250) in the culture medium. The purification was carried out using HiTrap-Protein G columns (GE Healthcare, 17-0404-01). The collected media was passed through the column and nonspecific binding was removed by washing with PBS. The elution was carried out using a 0.1M glycine-HCl buffer, pH 3.0. The purified IgGs were then dialyzed against PBS.

### ELISA and immuno blot analyses

ELISA plates (96 well, high bound Corning) were coated with 1 μg/ml active PDGF-CC, or recombinant PDGFRα protein (R&D, 6765-PR-050), in 50 μl 100 mM NaHCO_3_ (Sigma, S5761), at 4°C overnight. The plates were washed 3 times with PBS/0.1% Tween 20 (PBST), and then blocked with 3% bovine serum albumin (BSA: Saveen Werner AB, B 2000–500)/PBST at room temperature (RT) for 1 hour (h). The secreted supernatant from the hybridomas were subsequently applied as a dilution series starting from 1:2 in 3% BSA/PBS, then incubated for 1 h at RT. After washing, 50 μl goat anti-mouse IgG-AP (alkaline phosphatase) conjugated secondary antibody (Sigma, A3562) diluted 1:2500 in 3% BSA in PBS was applied for 1 h at RT. Bound antibodies were detected by developing with AP substrate containing *p*-nitrophenylphosphate (pNPP) (Sigma, P7998-100 ml), and the plates were read at 405 nm using the program Mars 1.11 in an ELISA reader (Spector star, Nano).

Immunoblots to detect the reactivity of human PDGF-CC GFD and full-length protein, mouse PDGF-CC (R&D, 1447-PC-CF), human PDGF-DD (R&D, 1159-SB-025/CF) against the mAbs were assessed as previously described [2].

### Neutralizing activity of the mAbs

PAE cells overexpressing PDGFRα (PAE-α) were seeded in Petri dish to 70% confluence. The day after, the cells were starved for 4 h in serum-free media at 37°C, washed with PBS, and stimulated with different amount of human core-domain PDGF-CC protein, or the mixture of PDGF-CC with different clones of mAb IgG using a 1:5 molar ratio. After 1 h stimulation on ice, the cells were washed, lysed and subjected to WB and PDGFRα tyrosine phosphorylation was assessed as previously described [8].

### Biosensor analysis—Affinity estimation

Biosensor analysis was performed on a BIAcore 2000 biosensor (GE Healthcare) using an NTA sensor chip as described in detail [33]. Briefly, a NTA sensor chip (GE Healthcare, BR1004-07) was loaded with Ni^2+^ and used to immobilize histidine tagged PDGF-CC ligand. The PDGF-CC antibodies were passed over the chip at varying concentrations in order to determine apparent affinity (K_D_). Chips were regenerated with ethylene glycol tetraacetic acid (EDTA, Sigma, E3889). The chip was re-equilibrated by washing with HBS (containing no EDTA) before further analysis. Using BIAevaluation software, a 1:1 Langmuir binding analysis model was used to estimate the apparent association (ka) and disassociation (kd) rate constants for each antibody generated (49).

### Biosensor analysis—Epitope determination

Competitive binding analysis was conducted for the panel of 4 anti-PDGF-CC candidate mAbs using immobilized PDGF-CC. The PDGF-CC antigen was covalently coupled to a CM5 Sensor Chip via primary amine groups using standard chemistry conditions of 0.05M NHS/0.2M EDC (49).

The binding ability of pairs of anti-PDGF-CC mAbs to bind simultaneously to immobilized PDGF-CC was tested. Monoclonal antibodies directed against separate epitopes will bind independently of each other, whereas antibodies directed against closely related epitopes will interfere with each other’s binding. Pair wise binding studies were performed by injection of the first antibody (50μl at the rate 100μl/ml) until surface saturation when all the available binding sites were occupied. Binding of the second antibody to PDGF-CC was then assessed following injection (50μl at the rate100μl/ml). Each analysis cycle was terminated by removing bound material from the sensor chip surface using 10mM Glycine, pH 2.1. Mapping was performed by analysing reciprocal duplicates of the same antibodies in reversed order. To further examine the nature of the binding to PDGF-CC protein and the potential for involvement of disulphide bonds within PDGF-CC, mAb binding was performed on non-reduced and reduced/alkylated antigen. Reduction and alkylation were performed on immobilized PDGF-CC by successive injection of 200μl of 50mM dithiothreitol (Sigma, 20–265) and 200μl of 30mM iodoacetamide (Sigma, l1149).

### Biosensor analysis-specificity of binding

Specificity of binding for PDGF-CC compared to the closely related PDGF-DD ligand was conducted. Both PDGF-CC and PDGF-DD (R&D, 1159-SB) antigens were immobilized to the biosensor chip via primary amine groups (NHS/EDC chemistry) using standard conditions. Anti-PDGF-CC mAbs (6B3, 11F5, 19C7, ch6B3) and a control anti-PDGF-DD mAbs were passed over the chip containing immobilized antigens.

### Immunohistochemistry—Tissue screens

Use of human tissue samples was approved by the Austin Hospital Human Research Ethics Committee. The identities of the patients or participants have been fully anonymized. Human tissue was provided by the Austin Hospital Department of Anatomical Pathology (Melbourne, Australia). Four micrometer paraffin-embedded sections were deparaffinized followed by quenching of endogenous peroxidase activity with 3% hydrogen peroxide (Merck, 107209) for 10 min at RT. Antigen retrieval was performed by boiling the sections in 10 mmol/l citric acid pH 6.0 for 30 min. Antibodies and dilutions used were the following: 6B3 (1.5 μg/ml), isotype control mouse IgG (Southern Biotechnologies; 1.5 μg/ml,) CD34 (0.5 μg/ml; Dako). Antibody binding was detected using Dako-Envision+ anti-mouse-HRP conjugated secondary antibody followed by DAB chromogen (Dako). As positive control sample the human lung carcinoma cell line A549 (ATCC) and positive placental tissue was used. Assay controls comprised replacing the primary antibody with an isotype control, or omission of the primary antibody. Test tissue was reported positive if a signal was present in the tissue incubated with antibody and if there was an absence of signal in sections incubated with the isotype control and/or sections incubated in the absence of primary antibody. In addition we assessed the specificity of 6B3 staining by using PDGF-CC ligand or the related growth factor VEGF-B as competitors. Murine 6B3 antibody (1.5 μg ml) was incubated with 6.7 X excess of PDGF-CC His6 antigen (10 μg/ml) or an excess of VEGF-B antigen (10 μg/ml, CSL) 1 h prior to antibody incubation to a section of A549 cell or human placental tissue. Placental tissue and A549 cell line sections reacted moderately to intensely (++ to +++) with the 6B3 antibody and the isotype and negative control sections were devoid of staining. In both ATCC and in human placental tissue, an excess of VEGF-B antigen did not interfere with 6B3 staining indicating that there is no non-specific staining to VEGF-B.

Slides were imaged using an Aperio ScanScope XT instrument (Aperio Technologies, Inc, Vista, CA) and viewed using ImageScope software (Aperio Technologies, Inc, Vista, CA). Anti-CD34 was applied to adjacent sections to easier judge PDGF-CC expression in blood vessels.

A modified histochemical score (H-score) was determined for each individual tissue sample based on the intensity of the staining and the percentage of positively stained cells [34]. This score was determined by multiplying the fraction of positive cells (range 0–5 [neg = 0; <5% = 1; 6–25% = 2; 26–50% = 3; 51–75% = 4; >75% = 5]) with the intensity of staining (neg = 0; + = 1; ++ = 2; +++ = 3). T-tests were conducted for each tissue type to determine statistical significance between 6B3 reactivity in control and tumour specimens. P value <0.05 was considered significant.

### Generation of chimeric anti-PDGF-CC antibody (ch6B3)

The cDNA sequences of murine monoclonal antibodies 6B3, 11F5, and 19C7 were determined using standard laboratory techniques. Based upon *in vitro* and *in vivo* characteristics, 6B3 was selected as a candidate for the generation of a mouse-human chimeric antibody and for future humanization.

Murine variable regions of the heavy chain (HC) and light chain (LC) clone 6B3 antibody were synthesized by GeneArt and cloned upstream of human IgG1 heavy and light chain kappa constant regions in pEE6.4 and pEE14.4 glutamine synthetase (GS) expression vectors (Lonza Biologics) respectively.

Following DNA sequence verification, the pEE6.4ch6B3HC and pEE14.4ch6B3LC vectors were digested with NotI/SalI restriction enzymes and the HC cassette from pEE6.4ch6B3HC was cloned into the pEE14.4ch6B3LC plasmid to make the double gene vector pDGVch6B3 as final construct. Expression using the GS system in both transient (Freestyle 293) and stable Chinese hamster ovary (CHO) cells was conducted and the PDGF-CC binding efficacy of ch6B3 was assessed using ELISA, Biacore and PDGF-CC neutralization activity *in vitro* as described previously for the murine monoclonal antibodies.

### Experimental animals

All experiments in this study were approved and performed in accordance with the guidelines from the Swedish National Board for Laboratory Animals under the ethical permit N85/08 that was approved by the North Stockholm Animal Ethics Committee. For the blood-retinal barrier leakage assay C57BL/6 mice were used. In addition we used *Pdgfrα H2B*-*eGFP* (*Pdgfrα*^*GFP/*+^) mice that express a nuclear GFP signal in cells, where the *Pdgfrα* promoter is/has been active [35].

### Blood-retinal barrier leakage assay

The blood-retinal barrier leakage assay was conducted using an established method [27]. Briefly, intraperitoneal injections with 400μg isotype control antibody, or ch6B3, were administrated to C57BL/6 mice (n = 4 for each group). 2 h post antibody treatment mice were put under light isoflurane anaesthesia and pupils were dilated by applying a drop of Mydriacyl (Apoteket, 043182) on top of the cornea. Under a stereomicroscope, a small incision was made in the sclera using a 25G needle and 500ng of PDGF-CC protein diluted in PBS (total volume 2μl) was carefully injected into the left eye behind the lens using a Hamilton syringe. A dose response with increasing concentrations of PDGF-CC was performed to identify the appropriate amount of PDGF-CC to be injected (data not shown). The same procedure was repeated for the right eye with injection of vehicle (2μl PBS). Subsequently, the 70 kDa tetramethyl rhodamine conjugated dextran (TMR-Dex) (Invitrogen, D34679) was injected intravenously in the tail vein. After 3 h of TMR-Dex circulation, mice were transcardially perfused with 60 ml HBSS (Invitrogen) and thereafter with 10 ml of 4% paraformaldehyde (PFA). Eyes were briefly post-fixed in 4% PFA for 30 min. Retina whole mounts were carefully dissected out and subjected to immunostaining and evaluation of permeability. Dextran and IF images were captured using a confocal microscope (Zeiss LSM700). Representative images shown are 2D renderings of 10 μm thick z-stacks. Fluorescence quantifications (pixel area) were performed using Image J software (NIH). The individual observations are based on analysis of five fields of vision from comparable anatomic positions.

### Immunostaining protocol

Whole mount retinas were permeabilized overnight in PBS/0.5%Triton-X100. Thereafter they were put in blocking solution o/n (TNB, Perkin Elmer, FP1012). The following day, Podocalyxin, (1:200, R&D, AF 1556), PDGFRα Cell Signaling, #3164) or GFAP (1:500, DAKO, Z0334) antibodies were applied in TNB overnight. After the primary antibody incubation, the retinas were washed in PBS/0.1% NP40 and this washing solution was exchanged 4 times in 1 day. Secondary Alexa488-conjugated anti-goat IgG antibodies (1:1000 in TNB, Invitrogen, A11055) were incubated overnight at 4 degree. Subsequently the retinas were thoroughly washed in PBS/0.1% NP40, exchanged 4 times in 1 day. DAPI (1:1000, Thermo Fisher, D1306) was added in one of the wash buffer exchanges. Incisions were made with a fine scissor to enable flat mounting with Vectashield (Invitrogen). Images were captured using an inverted epifluorescent microscope (Zeiss Observer, Zeiss) equipped with a 20X objective. Vascular permeability was assessed by evaluating the signal from the fluorescent TMR-Dex.

### Statistics

All statistical analyses were calculated using SPSS V18 (SPSS). Spearman rank correlation was used to determine the associations of PDGF-CC expression with clinical-pathological parameters.

## Results

### mAbs recognizing human PDGF-CC with high affinity

**Fig 1:**
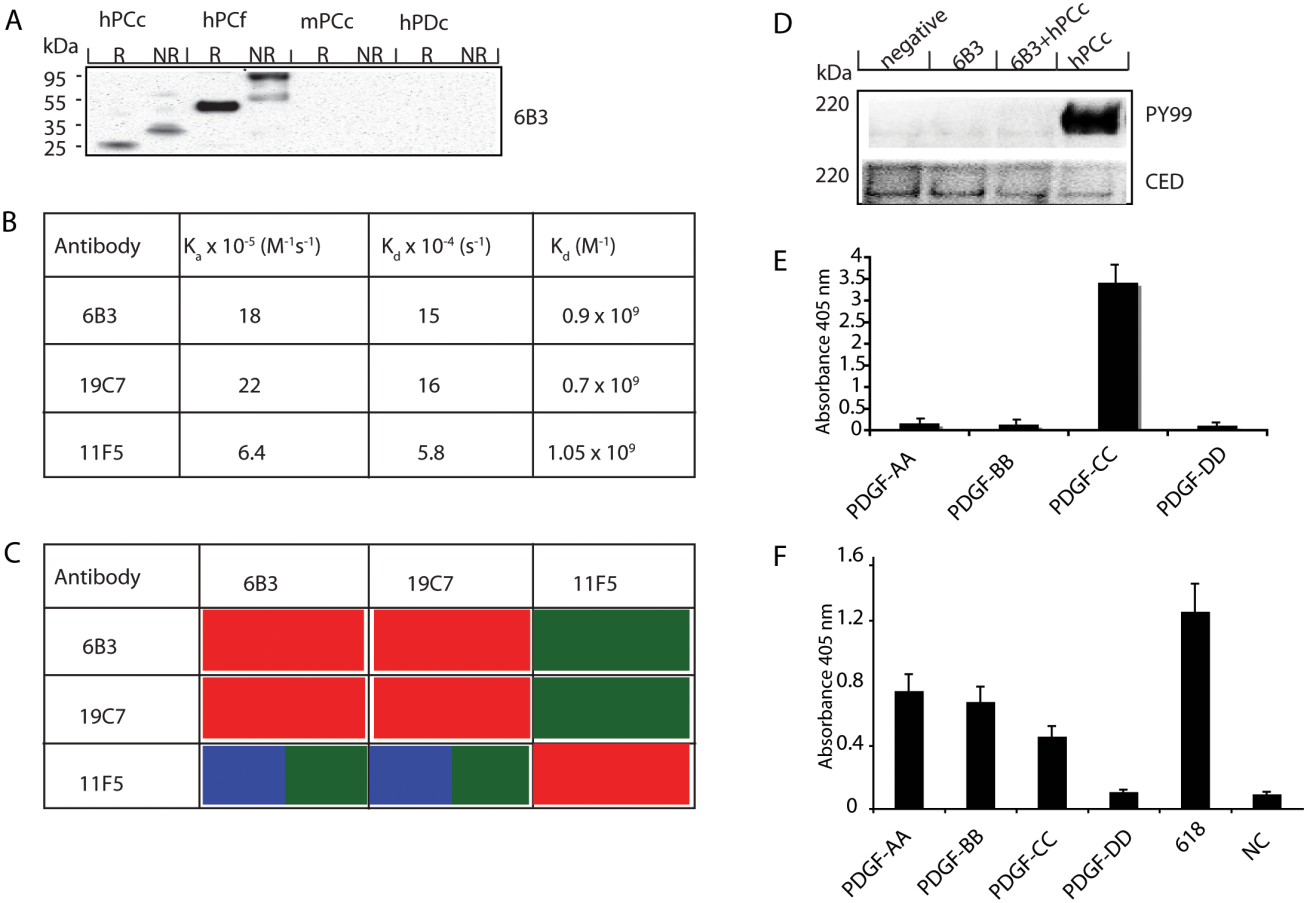
mAb 6B3 recognizes activated PDGF-CC with high affinity, and neutralizes PDGF-CC induced phosphorylation of PDGFRα. A) 6B3 recognizes the reduced (R) and non-reduced (NR) human PDGF-CC protein both the active (hPCc) and latent (hPCf) PDGF-CC in WB assay, but does neither recognize active (mPCc) mouse PDGF-CC, nor active human PDGF-DD (hPDc). B) Biosensor analysis of the apparent binding affinity. The apparent on-rate (ka), off-rate (kd) and dissociation constants are shown. The calculated dissociation constants for each antibody were in the low nano molar range, indicating that all 3 antibodies had a high affinity for the PDGF-CC ligand. C) Cross-competition for binding of mAbs to immobilized PDGF-CC. The effectiveness of the mAbs to cross compete for binding to immobilized PDGF-CC ligand is shown: Green: binding antibodies (different epitope); Red: competing antibodies (similar epitope); Blue: antibodies that interfere with binding. D) PAE1-PDGFRα cells were stimulated with either 30 ng hPCc, 6B3, or a mixture of 30 ng hPCc and preincubated 6B3 together for 1 h. PY99 was used to examine the phosphorylation status of PDGFRα. Only cells stimulated with PDGF-CC alone showed strong phosphorylation of PDGFRα, and this activation was blocked when 6B3 was pre-incubated together with the ligand (upper panel). The receptor expression level was detected with anti-CED (antibody against PDGFRs) and served as control. E) 96-well plate was coated with PDGF-AA, PDGF-BB, PDGF-CC or PDGF-DD and ELISA showed that 6B3 recognized only PDGF-CC but not other PDGF ligands. The value is the 405nM absorbance from the average of 8 samples ± standard deviation. F) Quality control for the PDGF ligands: 96-well plate was coated with recombinant PDGFRα protein or PDGF-DD and ELISA showed that PDGF-AA, PDGF-BB and PDGF-CC, but not PDGF-DD could detect PDGFRα. K618, a rabbit polyclonal antibody against PDGF-DD was used to assess protein quality of PDGF-DD. NC (not coated).

Screening of supernatants from generated hybridomas resulted in 16 clones containing IgGs with strong reactivity to PDGF-CC (data not shown). We selected stable and high IgG producing hybridomas, designated 6B3, 9A5, 11F5, 19A1, 19B1, 19C7 and 19D1 for further analysis (Table 1). IgGs from expanded cell culture supernatants were isolated and their specificity against human PDGF-CC, including cross-reactivity against mouse and rat PDGF-CC, were examined. Clone 6B3 recognized human latent and activated forms of PDGF-CC in both reduced and non-reduced conditions in immunoblots, but did not show cross-reactivity with mouse PDGF-CC. In addition, all other selected clones recognized human, but not mouse and rat PDGF-CC in immunoblots (Fig 1A and Table 1). Biacore analysis to determine the apparent on-rates (ka), off-rates (kd) and dissociation constants were performed to evaluate their binding affinities. The calculated dissociation constants for each antibody were in a low nanoMolar range, indicating that most of the antibodies have very high affinity for human PDGF-CC (Fig 1B). To further characterize the binding epitopes of the selected clones, we investigated the cross competition of mAbs for binding to immobilized PDGF-CC. Clones 6B3 and 19C7 demonstrated highly cross-competition while 11F5 showed no ability to compete with clone 6B3 and 19C7 (Fig 1C). This indicates that clone 6B3 and clone 19C7 have similar or overlapping epitopes, while 11F5 has a different epitope.

**Table 1:** Characteristics of the mAbs targeting PDGF-CC.

mAB name	Subclass	Isotype	Immunoactivity to mouse PDGF-CC	Neutralizing activity^a^
6B3 9A5 11F5 14D5 19A1 19B1 19C7 19D1	IgG2a IgG3 IgG2a IgG2b IgG3 IgG2a IgG2a IgG3	Kappa Kappa Kappa Kappa Kappa Kappa Kappa Kappa	No No No No No No No No	Strong Weak Strong Medium Weak Strong Strong Medium

IgG subtype classification, characterization of immunoreactivity to mouse PDGF-C and neutralizing activity of all antibodies analysed.

^a^ Neutralizing activity is determined by the capacity of the mAbs to block PDGF-CC induced phosphorylation of PDGFRα. Strong, medium or weak neutralizing activity implies that induction of PDGFRα phosphorylation with 60, 30 or 10 ng PDGF-CC, respectively, can be blocked in PDGFRα-expressing porcine aorta endothelia (PAE) cells.

We sequenced the CDRs of the variable heavy and light chains for 6B3, 19C7 and 11F5 using standard procedures (Table 2) [36]. Interestingly, the CDR sequences of 19C7 and 6B3 were identical, which explains the above-mentioned cross-competition results.

**Table 2:** Murine antibody isotypes and CDR sequence comparisons.

	**A. Variable light chain**		
	CDR1	CDR2	CDR3
6B3	KSSQSLLNSRNQKNYLA	FASTRES	QQHYSTPLT
11F5	KSSQSLLNSSNQKNYLA	FASTRDS	QQHYSTPLT
19C7	KSSQSLLNSRNQKNYLA	FASTRES	QQHYSTPLT
	**B. Variable light chain**		
	CDR1	CDR2	CDR3
6B3	GYTFRSYGIT	EIYPRSGKTYYNEKFKG	EGYGYDGGYFDY
11F5	GYIFISYGIS	EIYPRSGKTYYNEKFKD	EGYGYDGGYFDY
19C7	GYTFRSYGIT	EIYPRSGKTYYNEKFKG	EGYGYDGGYFDY

Sequencing of the CDR of the variable heavy and light chains for 6B3, 19C7 and 11F5 using standard procedures (Kabat combined with Chothia).

### Anti-PDGF-CC mAbs blocking phosphorylation of PDGFRα

To assess whether the characterized mAbs can neutralize PDGF-CC induced activation of PDGFRα, we analysed the capacity of the mAbs to block PDGF-CC induced receptor phosphorylation in PDGFRα-expressing porcine aorta endothelia (PAE) cells. Pre-incubation of activated human PDGF-CC with 6B3 IgG abolished phosphorylation of PAE-expressed PDGFRα, while an equivalent amount of the ligand alone lead to strong phosphorylation of the receptor (Fig 1D). Using the same experimental setting, we tested the neutralizing capacity of all other selected clones (Table 1). Our results indicated that we have generated 5 PDGF-CC neutralizing clones, namely 6B3, 11F5, 14D5, 19B1, and 19C7.

We tested 6B3 for potential cross-reactivity with the other 3 PDGF ligands, PDGF-AA, PDGF-BB, and PDGF-DD. We showed that 6B3 specifically recognized PDGF-CC without any cross-reactivity with the other PDGF ligands (Fig 1E). The same results were demonstrated using clones 11F5, 14D5, 19B1, and 19C7 (data not shown).

### Determination of the binding epitope for 6B3

**Fig 2:**
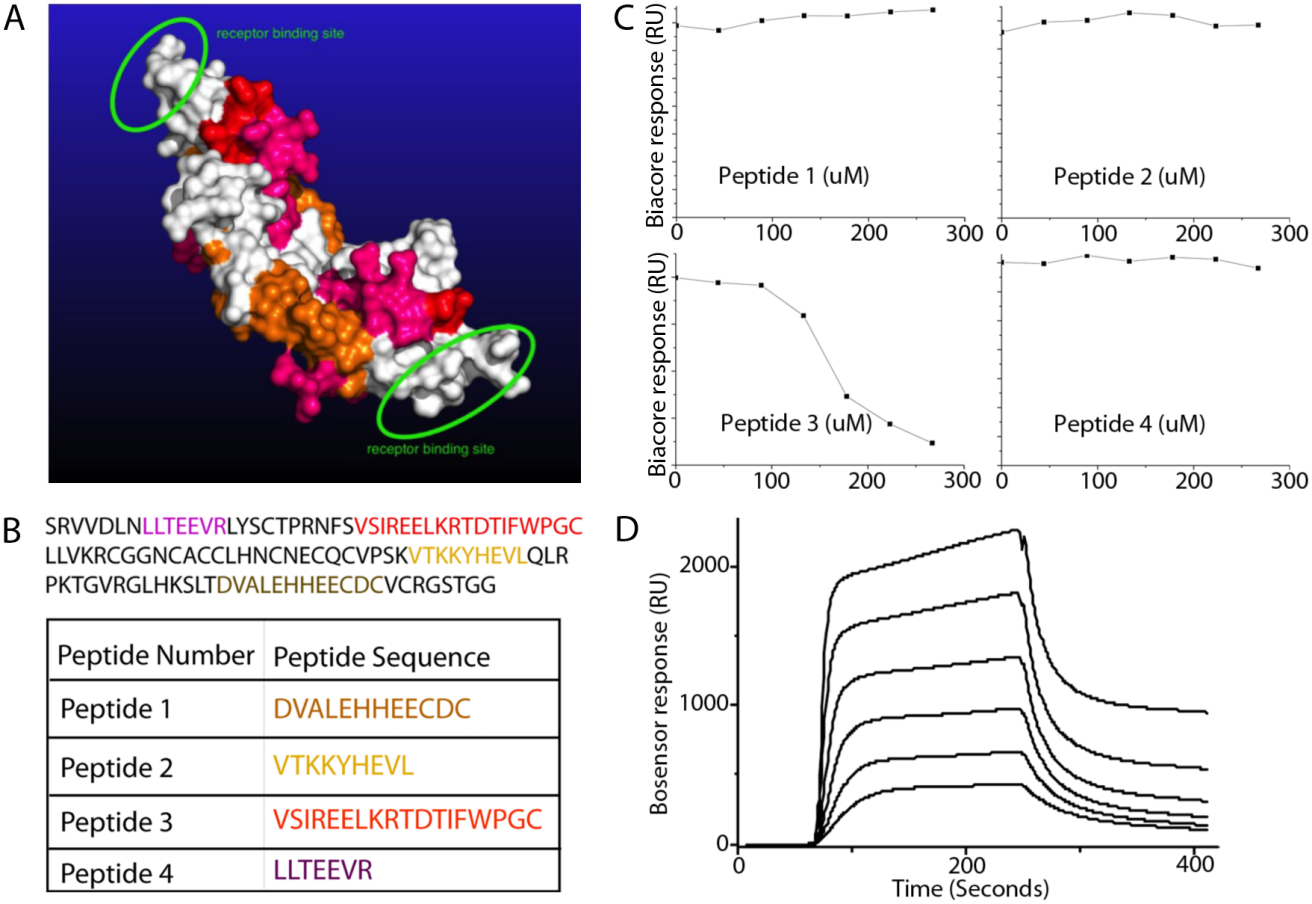
Computational predication and Biacore identification of the epitope for 6B3. A) Multiple Fragment Molecular Dynamics (MFMD) was used for modelling and prediction of the epitope for 6B3. The 3D structure of the activated PDGF-CC dimer showed 4 binding sites that are indicated with different colours. B) Amino acid sequence of activated PDGF-CC and the predicted peptides. C) Biocore analysis showed that peptide number 3 is competing with activated PDGF-CC for binding to 6B3 when 6B3 was pre-incubated with peptide 3, but not with any other peptides. D) Dose dependent binding of the immobilized peptide 3 to 6B3.

Epitope mapping of mAbs by a combination of CDR sequence determination and molecular modelling of the target antigen enables the visualization and localization of the key antigenic regions, and elucidates the structure-function relations of the mAb CDRs to the target antigen [37]. The interaction between activated PDGF-CC and the 6B3 antibody was predicted (Computist Bionanotech, Scoresby, Victoria) using the Multiple Fragment Molecular Dynamics (MFMD) method previously described [38] [39]. Four putative antigen-binding sites were predicted (Fig 2A). Accordingly, we designed and synthesized peptides corresponding to the 4 identified putative binding sites (Fig 2B). Biosensor chip analysis revealed that upon pre-incubation of 6B3 with peptide 3, the binding capacity of 6B3 for immobilized PDGF-CC was reduced in a dose-dependent manner (Fig 2C). This reduction was not detected with the other peptides. When peptide 3 was immobilized on the chip, application of increased concentration of 6B3 leads to increased binding (Fig 2D). These results confirmed that peptide 3 contains the epitope for 6B3.

### PDGF-CC is highly expressed in human normal and cancer tissues

**Fig 3:**
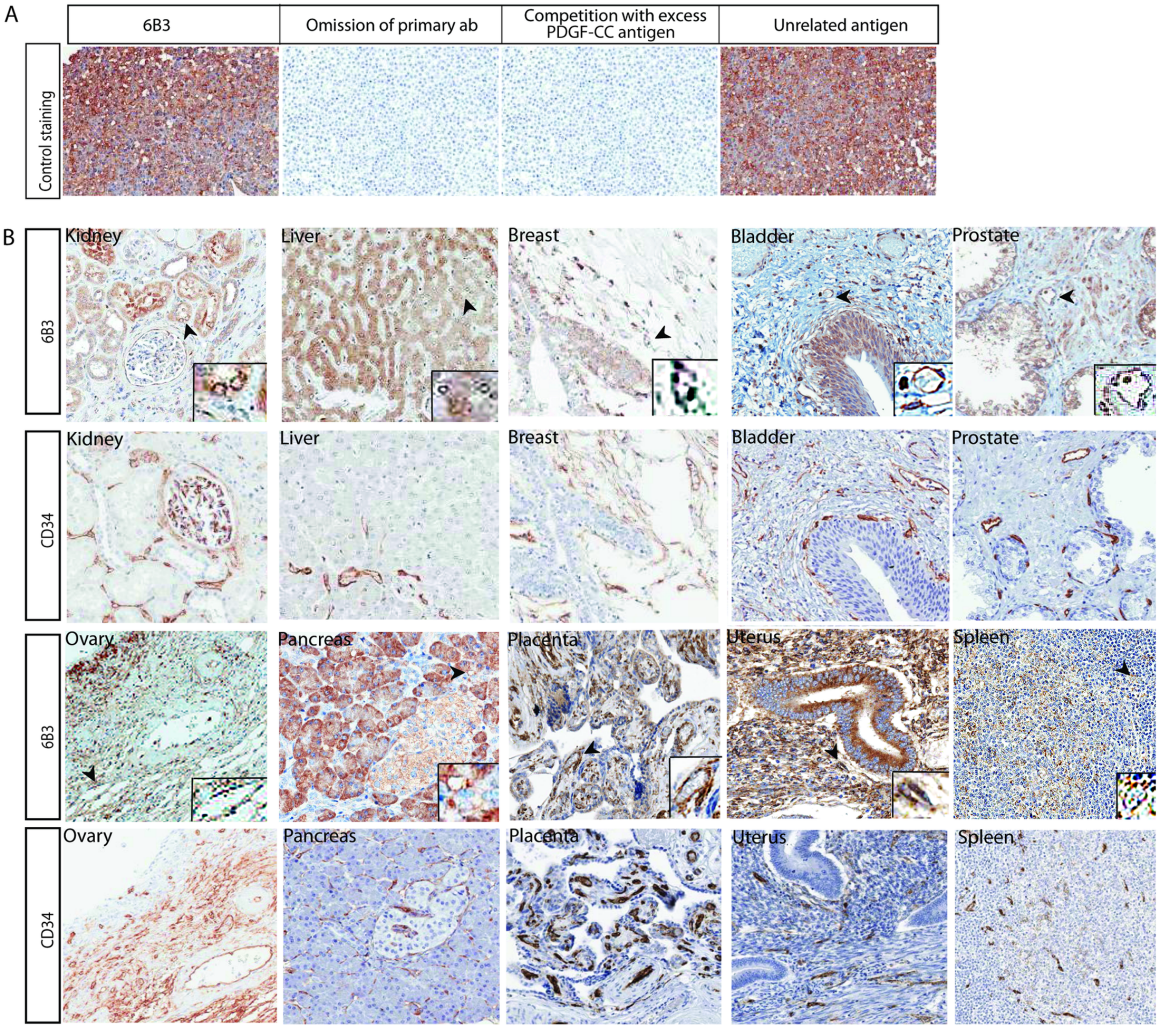
Analysis of PDGF-CC expression in human control tissue. Immunohistochemistry was used to localize PDGF-CC expression using 6B3 antibody in selected human control tissues. A) In the positive control (A459 cell line) staining with mAb 6B3 showed strong PDGF-CC expression (left panel) whereas no PDGF-CC expression could be detected upon omission of the primary antibody (2^nd^ panel to the right) or when the primary antibody was incubated with excess PDGF-CC protein (2^nd^ panel to the left), in contrast to incubation with an unspecific antigen (right panel). B) Representative images using 6B3 or CD34 antibodies in 10 control human tissues showed the differential expression level of PDGF-CC in various organs (see comments in Table 3). CD34 is used to show the outline of the blood vessels. Magnification 200X.

To explore whether the mAbs can be used as tools for monitoring expression of PDGF-CC in human tissues, we performed immunohistochemistry with 6B3 IgG in 17 control (Table 3) and 16 tumour tissues (Table 4). To evaluate the localization of PDGF-CC in blood vessels, we stained consecutive sections with the blood vessel marker CD34. The specificity of 6B3 was initially assessed using the cancer cell line A549, which previously has been shown to express high levels of PDGF-CC) [40]. Here mAb 6B3 showed strong PDGF-CC immunoreactivity. PDGF-CC expression could not be detected upon omission of the primary antibody or when the primary antibody was incubated with excess PDGF-CC protein, in contrast to incubation with an unspecific antigen (Fig 3A).

**Table 3:** Immunohistochemistry with anti-PDGF-CC antibody (6B3) in human control tissue.

Tissue type	Total	Epithelial cells	Stroma	Vessels	Comments
Adrenal	11/11	+ / ++	n/e	- / +++	adrenal medulla not sampled
Bladder, urinary	10/10	+ / +++	+ / +++	+ / +++	Urothelium
Bowel, large	12/12	-/+ / ++	+ / +++	++ / +++	incl. surface epithelium; lymphocytes within lamina propria
Brain	13/13	+ / +++	n/e	+ / ++	neurons/glia
Breast	7/7	+ / ++	- / +	+ / ++	myopeithelial cells
Heart	11/11	+	n/e	+ / ++	Myocardium
Kidney	12/12	+ / +++	n/e	+ / ++	parietal cells and endothelial cells in glomeruli (9/9)
Liver	13/13	++ / +++	-	- / ++	hepatocytes and bile ducts (11/11)
Lung	14/14	+ / ++	n/e	+ / ++	Type I and II pneumocytes
Ovary	10/10	+ / +++	++ / +++	+ / +++	follicular cysts and corpus luteum
Pancreas	12/12	+ / +++	n/e	+ / ++	Acini and Islets of Langerhan’s; Ducts (8/11)
Placenta	6/6	+ / ++	-	+++	Trophoblasts
Prostate	12/12	+ / +++	+ / +++	+ / +++	epithelial cells
Spleen	11/11	+ / +++	n/e	+ / +++	mainly positive staining in red pulp; stroma difficult to interpret
Thyroid	11/11	++ / +++	-	- / ++	follicular epithelium
Tonsil	12/12	+ / ++	n/e	++ / +++	germinal centres and lymphocytes
Uterus	3/3	++ / +++	+++	+ / ++	endometrial glands

17 human control tissues were stained with 6B3 and the evaluation was based on the uniformed scoring system for human healthy tissue. Abbreviations: n/e not evaluable or not evaluated; − negative; + weak staining; ++ moderate staining; +++ intense staining.

**Table 4:** Immunohistochemistry with anti-PDGF-CC antibody (6B3) in human tumour tissue.

Tumour type	Total	Tumour	Stroma	Vessels
Bladder TCC	10/10	++ / +++	n/e	++ / +++
Breast IDC	15/15	+ / +++	- / +++	+ / +++
Breast ILC	12/14	- / +++	++	++ / +++
Colorectal Adenocarcinoma	27/28	- / +++	+ / +++	+ / +++
Glioblastoma Multiforme	11/11	+ / +++	n/e	++ / +++
Gliosarcoma	9/9	++ / +++	n/e	++ / +++
Hepatocellular Carcinoma	16/16	++ / +++	n/e	+ / +++
Lung Adenocarcinoma	13/13	++ / +++	+ / ++	+ / +++
Lung SqCC	19/19	++ / +++	+ / +++	++ / +++
Metastatic Melanoma	19/19	+ / +++	n/e	+ / +++
Mesothelioma	11/11	+ / +++	+ / ++	+ / +++
Ovarian Adenocarcinoma	2/2	+++	n/e	++ / +++
Pancreatic Adenocarcinoma	8/8	+ / +++	++ / +++	++ / +++
Prostate Adenocarcinoma	13/13	++ / +++	+ / ++	+ / +++
Renal Cell Cancer	24/24	++ / +++	neg	+ / +++
Uterine Adenocarcinoma	8/8	++ / +++	+ / +++	+ / +++

16 tumour tissues were stained with 6B3 and the evaluation was based on the uniformed scoring system for human tumour tissue. Abbreviations: IDC infiltrating ductal carcinoma; ILC infiltrating lobular carcinoma; n/e not evaluable or not evaluated; neg negative; Sqcc squamous cell carcinoma; TCC transitional cell carcinoma. − negative; + weak staining; ++ moderate staining: +++ intense staining.

The results of the expression intensity and frequency of PDGF-CC stainings are based on the uniformed scoring system for human control (Table 3) and tumour tissue (Table 4). In control tissue, PDGF-CC was mainly observed in the cytoplasm of epithelial cells with an exception in ovaries, where it appeared in a membranous pattern (Fig 3B). We could detect PDGF-CC expression in blood vessels in all analysed organs (Fig 3B).

Cortical cells and the blood vessels of the adrenal gland exhibited weak to moderate cytoplasmic expression of PDGF-CC. In the bladder, PDGF-CC was observed in more than 50% of urothelium. In large bowel, the glands and epithelium generally showed a weak to moderate amount of PDGF-CC, whereas lamina propria exhibited increased expression intensity. The bile ducts exhibited weaker expression intensity (Table 3). In the brain, we observed weak to moderate neuronal and glial PDGF-CC expression. In breast tissue, PDGF-CC expression was predominantly observed in myoepithelial cells, also in the ductal cells, whereas fat cells showed weak expression.

Abundant expression of *Pdgfc* mRNA has been reported in human heart, liver, kidney, pancreas and ovaries, in contrast to other organs/tissues [2, 40]. In line with these reports, we detected abundant expression of PDGF-CC protein in the human breast, liver, kidney, pancreas, uterus, ovaries and prostate (Fig 3). In the human heart, we observed a weak expression of PDGF-CC in the myocardium and in some blood vessels (Table 3 and Fig 3). In the kidney, we detected widespread PDGF-CC expression in tubular epithelial cells and a weaker signal in the parietal cells of Bowmans capsule in the glomeruli. Moderate to strong expression intensity was observed in hepatocytes, again in concordance with a previous mRNA analysis [40]. In control pancreas, the acini exhibited a widespread moderate to strong expression intensity, in contrast to a weak expression in the ducts. In the thyroid gland, PDGF-CC was predominantly expressed in follicular epithelium and partially in vessels. Finally, in uterus, PDGF-CC was highly expressed throughout the whole organ (Table 3).

**Fig 4:**
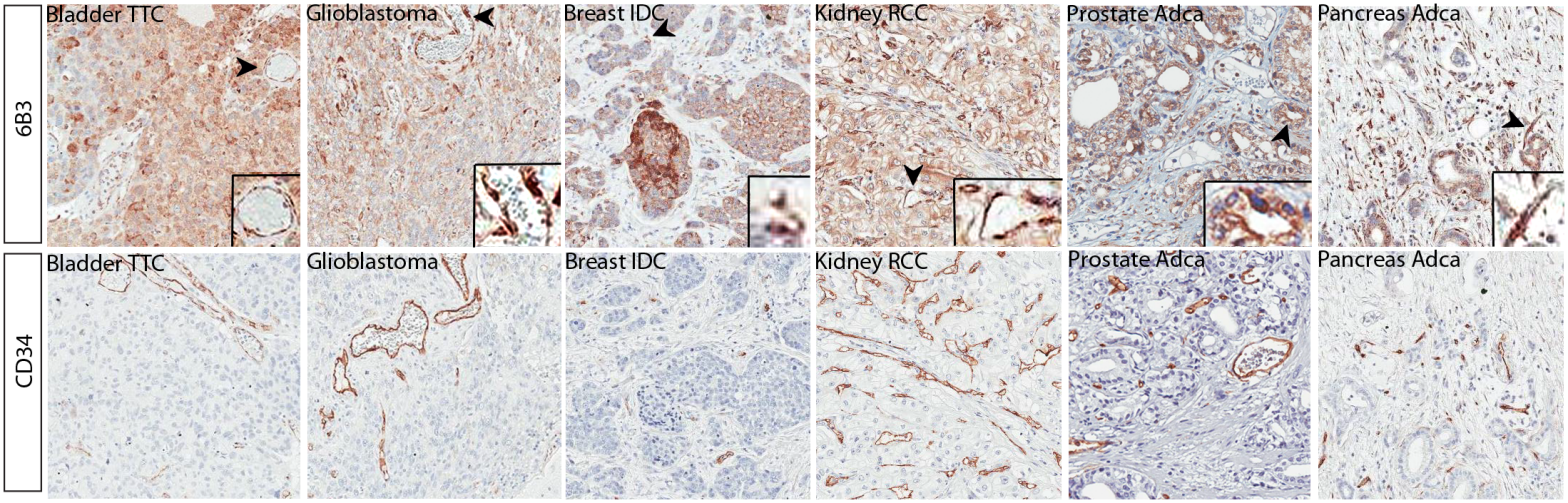
PDGF-CC expression is increased in various human tumour tissues in comparison to control tissues. Representative images using 6B3 and CD34 antibody in bladder transitional cell carcinoma (TCC), brain glioblastoma, breast infiltrating duct carcinoma (IDC), kidney renal cell carcinoma (RCC), pancreas and prostate adenocarcinoma (Adca) showed significantly higher expression level of PDGF-CC in comparison to their control tissues (see comments in Table 4), magnification 200X.

The expression pattern of PDGF-CC was also extensively investigated in various tumours (Fig 4). The protein was detectable in the cytoplasm of stromal compartments, in the cytoplasm and membrane of tumour cells, and in blood vessels. Notably, more than 75% of the tumours analysed showed moderate to strong PDGF-CC expression (Table 4). Control parenchyma including blood vessels had markedly decreased expression intensity compared to the respective tumour tissue (Table 5). Furthermore, the amount of positively stained cells was reduced in control compared to tumour tissue (Table 5). The difference in PDGF-CC expression between control and the respective tumour tissue was evaluated using a modified H-Score system [34] revealing increased PDGF-CC expression in epithelial tissues of bladder (p = 0.039), brain gliosarcoma (p<0.001), breast infiltrating ductal carcinoma (p = 0.006), renal cell cancer (p = 0.008), colon (p<0.001), pancreas (p<0.001) and prostate adenocarcinoma (p = 0.001) (Table 5).

**Table 5:** Organs with significant higher PDGF-CC expression in tumour than their respective control tissues.

Int	Bladder		Brain		Breast		Large Bowel	
	**Urothelium**	**Tumour**	**Neurons/Glia**	**Glio-sarcoma**	**Ducts/ Myoepithelial cells**	**IDC**	**Glands**	**AdenoCa**
Neg	0%	0%	0%	0%	0%	0%	0%	4%
+	30%	0%	31%	0%	43%	13%	83%	14%
++	30%	20%	61%	11%	57%	53%	17%	11%
+++	40%	80%	8%	89%	0%	34%	0%	71%
	**Stroma**				**Stroma**	**Stroma**	**Lamina Propria**	**Stroma**
Neg	0%				20%	15%	0%	0%
+	10%				80%	23%	8%	39%
++	10%				0%	53%	50%	50%
+++	80%				0%	9%	42%	11%
	**Vessels**	**Vessels**	**Vessels**	**Vessels**	**Vessels**	**Vessels**	**Vessels**	**Vessels**
Neg	0%	0%	0%	0%	0%	0%	0%	0%
+	20%	0%	85%	0%	86%	7%	42%	3%
++	70%	11%	15%	22%	14%	40%	50%	46%
+++	10%	89%	0%	78%	0%	53%	8%	51%

Tumour or control tissues were stained with 6B3 and the evaluation was based on the uniformed scoring system for human tumour and control tissue. The percentage of intensity of PDGF-CC staining in tumour and the respective control tissue is indicated. The percentage is indicated for negative, weak, moderate or intense PDGF-CC staining. Abbreviations: AdenoCa adenocarcinoma; IDC infiltrating ductal carcinoma; Int: Intensity of staining; n/e not evaluable or not evaluated; neg negative; + weak staining; ++ moderate staining: +++ intense staining.

### Characterization and functional application of chimeric 6B3

**Fig 5:**
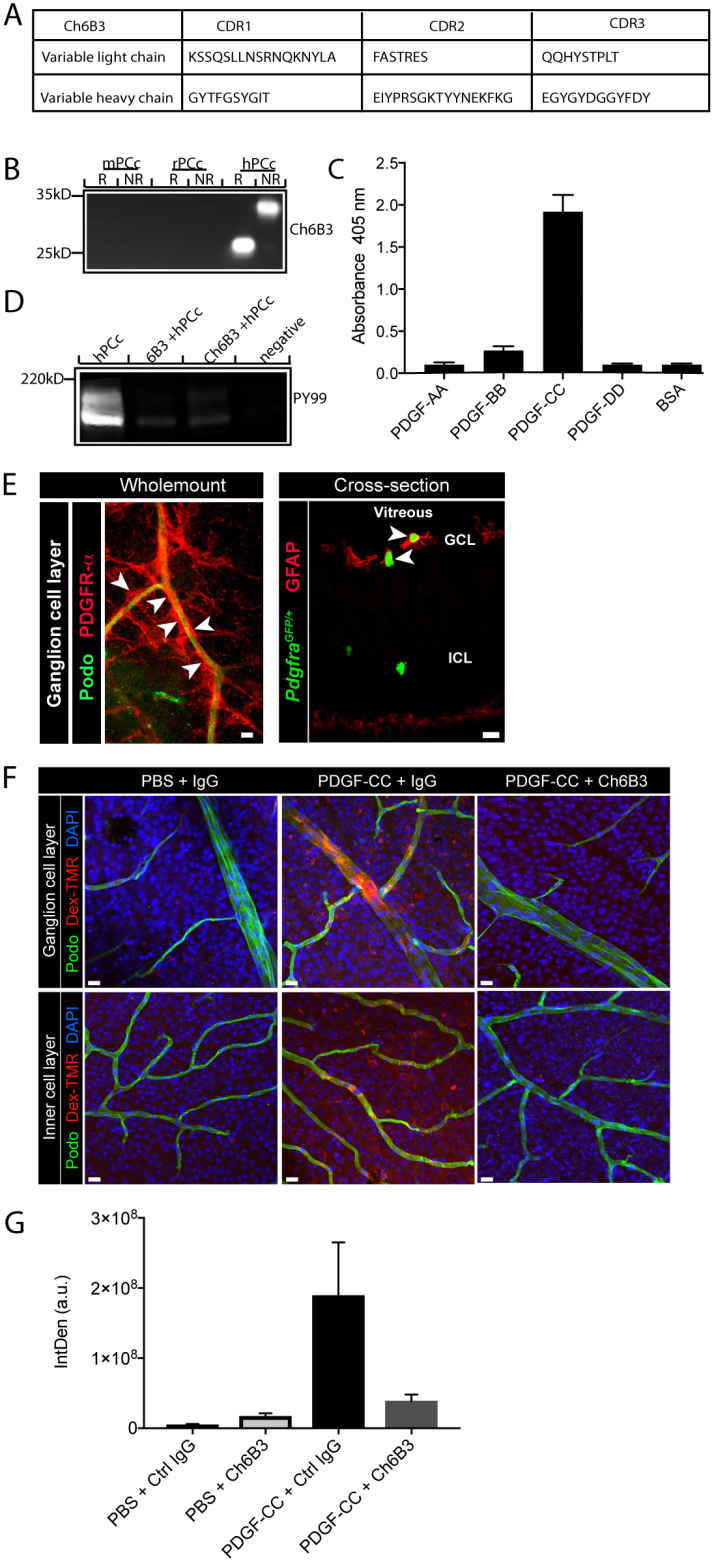
Structural and functional characterization of the chimeric 6B3. A) CDRs of the ch6B3 are indicated. B) Chimeric 6B3 (ch6B3) specifically recognizes hPDGF-CC under both reduced (R) and non-reduced (NR) conditions, but does not recognize mouse (mPDGF-CC) and rat (rPDGF-CC) PDGF-CC in immunoblots. C) ELISA showed that ch6B3 specifically detected PDGF-CC, but not any other PDGF ligands. D) Using PAE1-PDGFRα cells ch6B3 showed equal neutralizing capacity in comparison with mouse 6B3 in PDGF-CC induced phosphorylation of PDGFRα. E) Left panel: Co-staining for PDGFRα (in red) and the endothelial marker podocalyxin (in green) in whole-mount retina. Arrow heads point to perivascular PDGFRα+ cells ensheathing podocalyxin+ vessels (left panel). 50μm scale bar. Right panel: Co-staining for astrocyte marker GFAP (in red) in retina cross-section from *Pdgfrα*^*GFP/+*^ (in green) reporter mouse. Arrow heads point to double-positive cells in the ganglion cell layer, confirming that perivascular astrocytes in retina express PDGFRα 10 μm scale bar. F) Intraocular injection of PDGF-CC caused extravasation of the plasma-derived tracer TMR-Dex (red) in an *in vivo* BRB assay. BRB disruption was abrogated by intraperitoneal injection of ch6B3. Blood vessels were visualized by staining against Podocalyxin. Representative images are shown, based on n = 4 mice injected with isotype control Ig, and n = 4 mice injected with ch6b3 antibody. Scale bar 10 μm. G) Quantification of vascular permeability based on red fluorescent pixel area recorded in retina whole-mounts (n = 4 mice/experimental group). Abbreviations: hPCc: human PDGF-CC, GCL: ganglion cell layer, ICL: inner cell layer.

Mouse mAb chimerization is an important and powerful technique to reduce immunogenicity when using the murine mAbs in other species. We generated chimeric 6B3 (ch6B3) and characterized its biological and functional activity. We show that the CDR sequence of the generated ch6B3 differed only by one amino acid at the variable heavy chain in comparison to the original 6B3 (Fig 5A). Chimeric 6B3 recognized human PDGF-CC equally well in both reduced and non-reduced condition (Fig 5B). As expected, ch6B3 specifically binds to PDGF-CC but not to any other PDGF ligands (Fig 5C). To reveal whether it has the capacity to block PDGF-CC induced phosphorylation of PDGFRα, we performed a receptor stimulation assay using PDGFRα expressing PAE cells. Upon pre-incubation of ch6B3 with human PDGF-CC, we verified that the ligand was unable to induce phosphorylation of PDGFRα similar to what we observed with the original 6B3 mAb (Fig 5D).

We have previously demonstrated that PDGF-CC induces opening of the BBB *in vivo* [23]. To assess whether ch6B3 could be used *in vivo* for both functional assays and ultimately for development into a humanized mAb for therapeutic applications, we explored if ch6B3 could block PDGF-CC induced opening of the blood retinal barrier (BRB) following intraocular injection of active PDGF-CC into the vitreous of the mouse eye. The integrity of the BRB was assessed by injecting tetramethylrhodamine-labeled 70-kDa dextran (TMR-Dex) into mouse-tail vein. The BRB integrity was evaluated by measuring tracer extravasation into the perivascular tissue. Intraocular injection of active human PDGF-CC, but not PBS, induced extravasation of TMR-Dex from the blood vessels into the perivascular tissue (Fig 5E). In contrast, when ch6B3 was injected intraperitoneally prior to the intraocular injection of active human PDGF-CC, no extravasation of the tracer was observed (Fig 5E). Hence, a single injection of ch6B3 was sufficient to cross the compromised BRB and prevent PDGF-CC induced opening of the BRB.

## Discussion

Intercepting PDGF-CC signaling is a promising therapeutic avenue for targeting various types of cancer, fibrosis and neuropathologies involving BBB disruption [41, 42]. However, there are still no approved therapeutic monoclonal antibodies against PDGF-CC. We therefore aimed to develop both murine anti-human mAbs, and a chimeric anti-human mAb targeting PDGF-CC. We selected and extensively characterized the lead mAbs, 6B3 and ch6B3, that neutralize PDGF-CC-induced PDGFRα activation, and BRB disruption *in vivo*, in order to develop a potential novel agent against PDGF-CC-related malignancies and neuropathologies. This is to our knowledge the first study reporting specific mAbs against human PDGF-CC including *in vivo* assessment of their functionality.

The mAbs against PDGF-CC were generated from purified recombinant PDGF-CC protein. Specificity and binding affinity against the antigen have been evaluated. Although protein sequence of human and mouse PDGF-CC are 87% identical to (in the conserved GFD domain up to 90%) our mAb recognized human, and not mouse PDGF-CC in the assays we tested.

Despite the fact that PDGF-CC was discovered almost two decades ago, the detailed expression pattern in healthy and tumour tissue in humans has not been systematically reported yet. This is of utmost importance as PDGF-CC is crucial for tumour progression, angiogenesis and metastasis. We here used our novel mAb 6B3 to systematically screen various tumour and respective control tissues.

We assessed the expression pattern of PDGF-CC in human epithelial cells, stroma and in blood vessels (Tables 3 and 4). Notably, PDGF-CC was found in both epithelial and stromal cells, and showed a gradually increased expression upon tumour transformation. High *Pdgfc* mRNA [2] and PDGF-CC protein levels in the parietal epithelial cells of the Bowman's capsule, tubular epithelial cells, and in arterial endothelial cells were described previously using a polyclonal antibody [43]. Dijkmans et al. reported *PDGFC* mRNA expression in a range of human control tissues including heart, kidney, pancreas, small intestine, spleen, testes, thymus, brain, colon and lung [40]. However, they did not observe any obvious upregulation of *Pdgfc* mRNA in the tumour cell lines.

We could detect significantly increased PDGF-CC expression in stroma of both breast infiltrating duct carcinoma (IDC) (p = 0.039) and breast infiltrating lobular carcinoma (ILC) (p = 0.006) compared to control breast tissue. In addition, we observed a trend for increased PDGF-CC levels in stroma of prostate tumour (p = 0.08). Notably, PDGF-CC expression could not be evaluated in all tissue types due to the lack of stroma (Tables 3 and 4). In blood vessels significantly increased PDGF-CC levels were detected in bladder, brain, breast, colon, kidney, pancreas and prostate tumour biopsies compared to the respective control tissue (p<0.0001). Upregulation of PDGF-CC observed in the blood vessels indicates a potential role of this protein in tumour angiogenesis [44, 45]. In line with increased PDGF-CC expression in various tumours analysed in our study, it has been previously shown that PDGF-CC upregulation correlates with poorer prognosis of breast cancer, [46] colorectal cancer, [47] and glioma [48]. Moreover, activation of PDGF-CC promoted breast cancer metastases to bone [49]. Together with these observations, our data on PDGF-CC upregulation in the tumour samples strongly indicate relevance of targeting PDGF-CC for treating these pathologies.

We could detect increased PDGF-CC expression in breast infiltrating duct carcinomas (IDC) compared to breast infiltrating lobular carcinomas (ILC). Interestingly ILC tend to be slower growing and less aggressive than IDC and patients with ILC tend to have a better prognosis than those with IDC [50, 51]. In this respect it is interesting that pharmacological intervention of PDGF-CC activity with 6B3 in the MDA-MB-231 breast cancer model on SCID mice resulted in significant reduction of tumour size and micro-vascular density of about 30%. Most importantly 6B3 treatment led to conversion of poorly treatable basal-like breast cancers into a hormone receptor-positive state that enhanced sensitivity to endocrine therapy [52]. However PDGF-CC expression was not increased in glioblastoma, a very aggressive brain tumour [53]. A further analysis with a greater number of tumour samples together with relevant clinical information would be needed to explore the hypothesis whether PDGF-CC is higher expressed in more aggressive tumour types.

It has been demonstrated that overexpression of PDGF-CC results in liver fibrosis, steatosis and hepatocellular carcinoma in a *Pdgfc* transgenic mouse model [17]. As a majority of the control livers exhibited fibrosis or steatosis, we could not detect any statistical difference compared to the expression levels in hepatocellular carcinomas.

The BBB represents a dynamic interface between the central nervous system (CNS), the blood and the immune system. Since disruption of the BBB integrity is a common and significant event in the pathogenesis of several neurological disorders including stroke, multiple sclerosis, traumatic brain injury and Alzheimer’s disease, many efforts have been made for targeting mechanisms responsible for loss of the barrier integrity. We have previously reported that the PDGF-CC/PDGFRα axis controls the integrity of the BBB [23, 28]. Intraventricular injection of active PDGF-CC protein was sufficient to induce BBB opening [23]. Treatment with the small molecule inhibitor imatinib, which was shown to inhibit PDGFRs, was able to reduce BBB dysfunction in both acute and progressive experimental neuropathology models [23, 26–30]. Recently, we performed a phase II randomized trial with imatinib in patients with acute ischaemic stroke treated with intravenous thrombolysis [31]. We could show that imatinib significantly improved neurological outcomes with an improvement of 0.6 NIH stroke scale (NIHSS) points per 100 mg imatinib given. For the high dose group, the mean adjusted NIHSS improvements were even 5 points in comparison to controls.

Capillary loss and increased permeability of the BRB are among the earliest symptoms of diabetic retinopathy and treatment of the compromised BRB has been reported to ameliorate the symptoms [54]. We therefore aimed to determine the neutralizing capacity of the ch6B3 mAb *in vivo* using a BRB permeability assay. Indeed, intraocular PDGF-CC injection into the mouse eye induced disruption of the BRB, whereas intraperitoneal pretreatment with ch6B3 resulted in an intact BRB in contrast to the control retina injected with PBS (Fig 5E). The impact of PDGF-CC on vascular permeability, occurring within 1h, leads to increased leakage of antibodies from the blood stream out into the retina explaining the strong effect of the neutralizing antibody to inhibit vascular leakage, compared to the isotype control experiments. Thus, our data demonstrate high efficacy of circulating ch6B3 in blocking PDGF-CC-mediated PDGFRα signalling, indicating a possibility for *in vivo* targeting of PDGF-CC signalling for therapeutic purposes.
